# Cellular prion protein and NMDA receptor modulation: protecting against excitotoxicity

**DOI:** 10.3389/fcell.2014.00045

**Published:** 2014-08-28

**Authors:** Stefanie A. G. Black, Peter K. Stys, Gerald W. Zamponi, Shigeki Tsutsui

**Affiliations:** ^1^Department of Physiology and Pharmacology, University of CalgaryCalgary, AB, Canada; ^2^Hotchkiss Brain Institute, University of CalgaryCalgary, AB, Canada; ^3^Department of Clinical Neurosciences, University of CalgaryCalgary, AB, Canada

**Keywords:** NMDA receptor, cellular prion protein, excitotoxicity, neuroinflammation, ischemia, beta-amyloid, Alzheimer's disease

## Abstract

Although it is well established that misfolding of the cellular prion protein (PrP^C^) into the β-sheet-rich, aggregated scrapie conformation (PrP^Sc^) causes a variety of transmissible spongiform encephalopathies (TSEs), the physiological roles of PrP^C^ are still incompletely understood. There is accumulating evidence describing the roles of PrP^C^ in neurodegeneration and neuroinflammation. Recently, we identified a functional regulation of NMDA receptors by PrP^C^ that involves formation of a physical protein complex between these proteins. Excessive NMDA receptor activity during conditions such as ischemia mediates enhanced Ca^2+^ entry into cells and contributes to excitotoxic neuronal death. In addition, NMDA receptors and/or PrP^C^ play critical roles in neuroinflammation and glial cell toxicity. Inhibition of NMDA receptor activity protects against PrP^Sc^-induced neuronal death. Moreover, in mice lacking PrP^C^, infarct size is increased after focal cerebral ischemia, and absence of PrP^C^ increases susceptibility of neurons to NMDA receptor-dependent death. Recently, PrP^C^ was found to be a receptor for oligomeric beta-amyloid (Aβ) peptides, suggesting a role for PrP^C^ in Alzheimer's disease (AD). Our recent findings suggest that Aβ peptides enhance NMDA receptor current by perturbing the normal copper- and PrP^C^-dependent regulation of these receptors. Here, we review evidence highlighting a role for PrP^C^ in preventing NMDA receptor-mediated excitotoxicity and inflammation. There is a need for more detailed molecular characterization of PrP^C^-mediated regulation of NMDA receptors, such as determining which NMDA receptor subunits mediate pathogenic effects upon loss of PrP^C^-mediated regulation and identifying PrP^C^ binding site(s) on the receptor. This knowledge will allow development of novel therapeutic interventions for not only TSEs, but also for AD and other neurodegenerative disorders involving dysfunction of PrP^C^.

## Introduction

Misfolding of the cellular prion protein (PrP^C^) into the β-sheet-rich, aggregate-prone scrapie conformation (PrP^Sc^) is well known to result in several progressive and often fatal diseases termed prionopathies. This group of disorders, also known as transmissible spongiform encephalopathies (TSEs), includes scrapie, bovine spongiform encephalopathy (BSE, or mad cow disease), and the human diseases Creutzfeld–Jakob disease, Gerstmann-Straussler-Scheinker syndrome, fatal familial insomnia, and Kuru (Aguzzi et al., 2008). PrP^Sc^ aggregation and formation of amyloid-like plaques disrupt neuronal physiology, and most TSEs involve eventual loss of neurons (Aguzzi et al., 2008). Although much work has been done investigating the deleterious effects of misfolded/aggregated prion proteins, the physiological roles of PrP^C^ remain incompletely understood.

**Figure 1:**
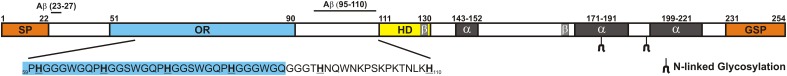
**Schematic representation of PrP^C^**. Numbering of amino acid residues corresponds to the mouse PrP^C^ sequence. Shown are the locations of histidine (H) residues that bind copper ions (residues 60, 68, 76, 84, 95, 110), the amino (N)-terminal signal peptide (SP; residues 1–22), β-amyloid oligomer (Aβ) binding regions (residues 23–27, 95–110), the octapeptide repeat region (OR; residues 51–90), the central hydrophobic domain (HD; residues 111–130), α-helical regions (α; residues 143–152, 171–191, 199–221), β-sheet regions (β; residues 127–129, 166–168), sites of asparagine (N)-linked glycosylation (residues 180, 196), and the carboxyl (C)-terminal glycosylphosphatidylinositol (GPI) signal peptide (GSP; residues 231–254).

Mature PrP^C^ is a glycoprotein that is anchored to the extracellular leaflet of the plasma membrane through a carboxyl (C)-terminal glycosylphosphatidylinositol (GPI) anchor. Initially, PrP^C^ is synthesized as a precursor polypeptide (254 amino acids for mouse PrP^C^) containing an amino (N)-terminal signal sequence that directs PrP^C^ to the endoplasmic reticulum and is post-translationally cleaved, and a C-terminal GPI anchor sequence that is removed and replaced with a GPI anchor (Figure 1; Biasini et al., 2012). The N-terminal portion of PrP^C^ is intrinsically unstructured, contains the octarepeat region, and has multiple copper binding sites both within and outside of the octarepeat region (Figure 1; Klewpatinond et al., 2008; Stanyon et al., 2014). The PrP^C^ N-terminus also contains sites for binding of oligomeric β-amyloid (Aβ) peptides (Figure 1; Lauren et al., 2009; Chen et al., 2010). In contrast to the N-terminus, the C-terminus of PrP^C^ is globular in structure, with three α-helical and two short β-strand regions, and is the location of two asparagine residues that undergo glycosylation (Figure 1; Biasini et al., 2012). In addition, PrP^C^ can undergo physiological cleavage of different types: α-cleavage in the vicinity of residue 109 (mouse sequence) generates N1 and C1 fragments; β-cleavage in the vicinity of residue 90 (mouse sequence) produces N2 and C2 fragments; and cleavage at the GPI anchor releases full-length PrP^C^ into the extracellular environment (Altmeppen et al., 2013; McDonald et al., 2014). However, recent data from McDonald and co-workers using recombinant mouse PrP^C^ reveal that cleavage of PrP^C^ is quite complex, generating a number of different N1, C1, N2, and C2 fragments (McDonald et al., 2014), although the biological functions of these cleavage products remain to be determined.

PrP^C^ interacts with and signals through many different cell surface proteins, including the α7 nicotinic acetylcholine receptor (Beraldo et al., 2010), metabotropic glutamate receptors mGluR1 (Beraldo et al., 2011) and mGluR5 (Beraldo et al., 2011; Um et al., 2013), kainate receptor GluR6/7 (Carulla et al., 2011), and α-amino-3-hydroxy-5-methyl-4-isoxazole propionic acid (AMPA) receptor subunits GluA1 (Watt et al., 2012) and GluA2 (Kleene et al., 2007; Watt et al., 2012). Furthermore, Aβ oligomers can bind to PrP^C^ and signal through mGluR5 to activate Fyn kinase (Um et al., 2013), and an Aβ-PrP^C^-mGluR5 pathway is also involved in facilitation of long-term depression (LTD) (Hu et al., 2014). The diversity of signaling mediated by PrP^C^ highlights the need for a better understanding of how the interaction of PrP^C^ with its binding partners is regulated in both physiological and pathological conditions. Work from our laboratory revealed that PrP^C^ also interacts with N-methyl-D-aspartate (NMDA) receptors; PrP^C^-deficient mice display increased NMDA receptor-dependent neuronal excitability and are more susceptible to NMDA-induced excitotoxicity (Khosravani et al., 2008). Here, we briefly review properties of the NMDA receptor and focus on evidence supporting a role for PrP^C^ in preventing NMDA receptor-mediated excitotoxicity, a process that may contribute to the pathogenesis of a variety of disorders including ischemic stroke, Alzheimer's disease (AD), Huntington's disease, and epilepsy (Lai et al., 2014; Parsons and Raymond, 2014). Similar mechanisms may be involved in PrP^Sc^-induced toxicity (Müller et al., 1993; Riemer et al., 2008; Resenberger et al., 2011).

## NMDA receptor characteristics

Glutamate is the major excitatory transmitter in the mammalian central nervous system (CNS), and it exerts its actions by binding to a variety of different receptor proteins. Glutamate receptors (GluRs) are divided into two families, ionotropic and metabotropic. Metabotropic GluRs are G protein-coupled receptors, while ionotropic GluRs are ion channels. There are three types of ionotropic GluRs, namely AMPA, kainate, and NMDA receptors (Mayer, 2005).

NMDA receptors are cation channels that mediate entry of Na^+^ and Ca^2+^ ions and are activated by the co-agonists glutamate (or NMDA) and glycine or D-serine. Activation of NMDA receptors thus contributes to the excitatory postsynaptic potential (EPSP), as well as both long-term potentiation (LTP) and LTD (Bartlett and Wang, 2013). In addition to co-agonist binding, activation of NMDA receptors requires membrane depolarization to remove the Mg^2+^ ion block of the channel pore that occurs at resting membrane potentials. NMDA receptors are heterotetrameric channels formed by the assembly of two obligatory GluN1 and two GluN2/GluN3 subunits. GluN1 subunits contain the binding site for glycine, while glutamate binds to GluN2 subunits. To date, there are seven known NMDA receptor subunits: GluN1, GluN2A-GluN2D, and GluN3A-GluN3B, with alternative splicing of GluN1 and GluN3 subunits generating further diversity (Paoletti et al., 2013). GluN3 subunits also bind glycine, and thus NMDA receptors containing only GluN1 and GluN3 subunits give rise to “glycine-only” receptors that cannot be activated by glutamate or NMDA (Chatterton et al., 2002; Pina-Crespo et al., 2010). Identity of the GluN2 subunits that combine with GluN1 subunits to form the tetrameric channel dictates NMDA receptor properties, such as activation and deactivation kinetics, ion conductance, and affinity for glutamate (Cull-Candy and Leszkiewicz, 2004). Pharmacological characterization using ligands for specific GluN2 subunits allows differentiation of receptor subtypes (Wyllie et al., 2013).

NMDA receptor localization can be synaptic, perisynaptic, extrasynaptic, or even presynaptic, with receptor activity at each location coupling to specific cellular events (Corlew et al., 2008; Hardingham, 2009; Hardingham and Bading, 2010; Paoletti et al., 2013). In general, activation of synaptic NMDA receptors activates pro-survival signaling, while extrasynaptic NMDA receptor activity mediates pro-death signaling (Hardingham, 2009; Hardingham and Bading, 2010). Thus, while NMDA receptors mediate key physiological functions such as learning and memory under normal conditions, they also play a role in glutamate excitotoxicity, which can occur in response to ischemia (Lau and Tymianski, 2010) and is involved in many neurodegenerative conditions, including AD (Parsons and Raymond, 2014). In these abnormal situations, excessive glutamate can spill over from synaptic to extrasynaptic sites, thus activating not only the desired synaptically-localized NMDA receptors, but also those receptors located extrasynaptically. Because NMDA receptors are highly permeable to Ca^2+^ ions, this overstimulation leads to enhanced Ca^2+^ influx that ultimately can be fatal to cells (Lau and Tymianski, 2010). NMDA receptors are found not only in neurons, but also in oligodendrocytes and myelin, and ischemia can cause NMDA receptor-dependent damage to both myelin and oligodendrocyte processes (Karadottir et al., 2005; Salter and Fern, 2005; Micu et al., 2006). Hence, tight regulation of NMDA receptor activity is of extreme importance in maintaining physiological signaling while preventing excitotoxicity. NMDA receptor activity is regulated by a number of different mechanisms, one of which involves an intrinsic desensitization mechanism that results in termination of channel activity even in the prolonged presence of glutamate, a process that is potently regulated by the receptor co-agonist glycine (Mayer et al., 1989). Recently, we demonstrated that the absence of PrP^C^ enhances NMDA receptor glycine affinity, leading to persistent NMDA receptor activity upon prolonged agonist application (You et al., 2012), which likely underlies the greater susceptibility of PrP^C^-null neurons to NMDA receptor-mediated damage and dysfunction (Rangel et al., 2007; Khosravani et al., 2008; Gadotti and Zamponi, 2011; Gadotti et al., 2012; You et al., 2012; Fleisch et al., 2013).

## PrP^C^ as a regulator of NMDA receptors

In support of a role for PrP^C^ in preventing NMDA receptor hyperactivity, data from our and other laboratories reveal that NMDA receptor activity is enhanced when PrP^C^ is absent. Our work using mouse hippocampal slices showed that the enhanced neuronal excitability in PrP^C^-null neurons can be reduced by the NMDA receptor blocker aminophosphonovaleric acid (APV), and that NMDA receptor-mediated miniature excitatory postsynaptic currents (mEPSCs) recorded from cultured hippocampal neurons from PrP^C^-null mice had significantly larger amplitudes and longer decay times than those recorded from wild-type neurons (Khosravani et al., 2008). In this study we also showed that NMDA application resulted in whole-cell currents with a prolonged time course of deactivation in cultured PrP^C^-null hippocampal neurons that could be restored to wild-type levels by ectopic expression of mouse PrP^C^ and mimicked by reducing PrP^C^ expression in cultured wild-type hippocampal neurons. Co-immunoprecipitation experiments demonstrated that PrP^C^ and GluN2D subunits were found in the same protein complex, and immunofluorescent staining of hippocampal neurons showed co-localization of these two proteins. Indeed, the properties of the NMDA-evoked currents we observed in PrP^C^-null neurons in this study appeared to resemble those of heterologously-expressed GluN2D-containing NMDA receptors (Cull-Candy and Leszkiewicz, 2004). Furthermore, siRNA knockdown of GluN2D expression dramatically accelerated NMDA receptor current deactivation kinetics and reduced current amplitude. Taken together, these observations point to an upregulation of GluN2D-containing NMDA receptor activity in the absence of PrP^C^. However, it is also possible that activity of NMDA receptors containing other subunits is altered by the lack of PrP^C^, producing GluN2D-like deactivation kinetics. Finally, in support of a role for PrP^C^ in protecting against NMDA receptor-mediated toxicity, we observed significantly more cell death in response to NMDA treatment of PrP^C^-null hippocampal cultures than in wild-type cultures, and that focal injection of NMDA into the hippocampus resulted in a lesion of significantly greater size in PrP^C^-null mice than in wild-type mice (Khosravani et al., 2008). This increase in NMDA receptor activity not only gives rise to an increased susceptibility of neurons to cell death, but also affects other physiological processes that are linked to NMDA receptor function. For example, NMDA receptors are important for the transmission of pain signals at the spinal level. Consequently, in models of inflammatory and neuropathic pain, we found enhanced nociceptive responses in PrP^C^-null mice that could be restored to control levels by MK-801 (Gadotti and Zamponi, 2011). It is also known that NMDA receptors play a role in depressive-like behavior at the level of the hippocampus. Consistent with this notion, we found that PrP^C^-null mice displayed increased depressive-like behavior that could be abrogated by NMDA receptor blockers (Gadotti et al., 2012).

It is well established that PrP^C^ is a copper-binding protein, with up to six copper ions binding to histidine residues in the N-terminal region both within (mouse PrP^C^ residues 60, 68, 76, and 84) and outside of (mouse PrP^C^ residues 95 and 110) the octarepeat region (Figure 1; Klewpatinond et al., 2008; Quintanar et al., 2013). In addition, copper may bind to histidine residues 176 and 186 (mouse sequence) in the C-terminus of PrP^C^ (Quintanar et al., 2013), and recent evidence shows that copper can bind to the N-terminal amino group of PrP^C^ (Stanyon et al., 2014). Occupancy of these copper sites is known to alter PrP^C^ structure (Qin et al., 2000; Thakur et al., 2011; Younan et al., 2011; Quintanar et al., 2013), and thus likely its interactions with other protein partners. The ability to examine NMDA receptor activity as an indirect readout of the interaction of the receptors with PrP^C^ has provided interesting insights into the roles of the copper binding sites for PrP^C^ function. Under normal circumstances, NMDA receptors undergo desensitization in the prolonged presence of glutamate or NMDA, and this process is modulated by the co-agonist glycine (Mayer et al., 1989). In cultured mouse hippocampal neurons lacking PrP^C^, the desensitization kinetics appear to be slowed such that a non-desensitizing current is observed (You et al., 2012). In wild-type neurons, copper chelation using bathocuproine sulfonate (BCS) or cuprizone induced a persistent NMDA current similar to that seen in PrP^C^-null neurons (You et al., 2012). Acute treatment with phosphatidylinositol-specific phospholipase C (PI-PLC), which enzymatically removes proteins such as PrP^C^ that are linked to the extracellular leaflet of the plasma membrane via GPI anchors, also induced a persistent NMDA-dependent current in rat hippocampal neurons that was similar to both the current seen with copper chelation and to that recorded from PrP^C^-null mouse hippocampal neurons (You et al., 2012). Moreover, PI-PLC treatment did not alter the steady-state NMDA current in PrP^C^-null neurons, indicating that the enzymatic cleavage did not measurably affect other potential regulatory proteins (You et al., 2012). Experiments investigating the effect of glycine concentration on steady-state NMDA current revealed that at a given glycine concentration, a higher level of steady-state current was seen in neurons lacking PrP^C^ than in wild-type neurons; a similar effect was seen upon copper chelation (You et al., 2012). In rat hippocampal cultures, copper chelation induced cell death that could be prevented by NMDA receptor inhibition or re-addition of excess copper (You et al., 2012). Taken together, these results indicate that copper ions modulate NMDA receptors by virtue of their interactions with PrP^C^. Furthermore, these findings suggests that copper ions are key regulators of NMDA receptor function, thus adding another layer of metal ion regulation of these receptors in addition to the well described role of zinc ions.

Further support for PrP^C^-mediated regulation of NMDA receptor activity comes from a study using zebrafish with a targeted mutation of the *prp2* gene encoding the zebrafish prion protein PrP2, which has characteristics similar to mammalian PrP^C^ (Fleisch et al., 2013). Consistent with observations in mammalian brain, these mutants displayed increased convulsant-induced seizure activity compared to wild-type animals. Analysis of mEPSCs recorded *in vivo* from intact zebrafish hindbrain neurons showed that the frequency of NMDA receptor-mediated mEPSCs was reduced in *prp2*^−/−^ fish compared to controls, and although NMDA receptor current amplitudes were the same in both groups, NMDA receptor currents had longer decay times in *prp2*^−/−^ zebrafish. These recent observations agree with the earlier findings of Walz and colleagues showing that mice lacking PrP^C^ were more sensitive to convulsant-induced seizures, and significantly more PrP^C^-null mice died than wild-type mice (Walz et al., 1999). However, a recent study found elevated thresholds for convulsant-induced epileptiform activity in hippocampal slices from PrP^C^-null mice compared to wild-type controls (Ratte et al., 2011). The reason for this disparity in the literature remains to be determined.

The presence of misfolded prion proteins could induce NMDA receptor hyperfunction by preventing normal, PrP^C^-mediated control of NMDA receptor activity. Indeed, there are several examples of augmented NMDA receptor function in the presence of mutant or scrapie forms of the prion protein. It has been demonstrated that NMDA receptor antagonists memantine and MK-801 prevented PrP^Sc^-induced toxicity in cultured rat cortical neurons (Müller et al., 1993). Memantine also delayed death of scrapie-infected mice (Riemer et al., 2008) and blocked the PrP^Sc^-induced increase in apoptosis of PrP^C^-expressing SH-SY5Y neuroblastoma cells (Resenberger et al., 2011). Recently, Biasini and co-workers revealed a role for aberrant NMDA receptor activity in the toxic effects of a prion protein lacking residues 105–125 in the central region, termed ΔCR PrP (Biasini et al., 2013). In this study, organotypic cerebellar slice cultures from ΔCR PrP-expressing mice were more susceptible to glutamate-, kainate-, and NMDA-induced cell death compared to those from PrP^+/−^ controls, an effect that was rescued by overexpression of wild-type PrP^C^. Notably, NMDA had a more marked effect on cell death compared to the other treatments. These authors also observed that ΔCR PrP expression induces spontaneous inward currents, which may contribute to enhanced NMDA receptor activity via depolarization-induced relief of the Mg^2+^ ion block of these receptors. Importantly, the toxic NMDA receptor-dependent effects of ΔCR PrP in this study were prevented by overexpression of wild-type PrP^C^, again supporting a neuroprotective role of PrP^C^ via prevention of NMDA receptor-mediated toxicity. In another recent study, the toxicity of the misfolded form of a prion protein fragment comprised of residues 90–231 was found to be mediated in part by NMDA receptor-dependent excitotoxicity (Thellung et al., 2013). In this study, treatment of cerebellar granule neuron cultures for 1–3 days with the misfolded prion protein 90–231 fragment augmented both intracellular Ca^2+^ levels and apoptosis compared to that measured in control cells, effects that were nearly completely abolished by NMDA receptor blockade.

Altogether, the data obtained from studies using PrP^C^-null animals suggest that there is aberrant NMDA receptor activity in the absence of normal PrP^C^ function, which is further supported by the fact that exogenous expression of PrP^C^ can prevent the NMDA receptor-dependent toxic effects of misfolded or mutant prion proteins. This raises the possibility of restoring PrP^C^-mediated regulation of NMDA receptors in pathologies where PrP^C^ function is perturbed as a therapeutic intervention to control NMDA receptor-mediated toxicity. This could in theory be accomplished by mimicking the interaction of PrP^C^ with NMDA receptors. To achieve this, more details regarding the molecular determinants governing the interaction of PrP^C^ and NMDA receptor proteins are required. Additionally, if loss of physiological PrP^C^ function results in augmented activity of certain NMDA receptor subtypes, such as those containing GluN2D subunits, a thorough molecular characterization of the PrP^C^-NMDA receptor interaction will allow development of targeted pharmacotherapies to reduce hyperfunction of only those receptors affected whose activity is aberrantly upregulated. This approach could prevent undesired effects of blocking physiological NMDA receptor signaling.

## Absence of PrP^C^ exacerbates ischemic injury—a role for NMDA receptor hyperactivity?

Given the neuroprotective effects of PrP^C^, it may not be surprising that absence of PrP^C^ can exacerbate the neuronal damage that ensues following an ischemic insult. Several studies have demonstrated increased ischemic brain damage in PrP^C^-null mice (McLennan et al., 2004; Sakurai-Yamashita et al., 2005; Spudich et al., 2005; Weise et al., 2006; Mitteregger et al., 2007; Steele et al., 2009). However, ischemia can induce an upregulation of PrP^C^ mRNA (McLennan et al., 2004; Mitsios et al., 2007; Mitteregger et al., 2007) and protein (McLennan et al., 2004; Weise et al., 2004; Shyu et al., 2005; Adle-Biassette et al., 2006; Mitsios et al., 2007; Mitteregger et al., 2007), presumably as a compensatory protective mechanism. The idea of compensatory upregulation of PrP^C^ expression protecting against cell death in ischemia is supported by the findings that adenovirus-mediated overexpression of PrP^C^ reduced ischemia-induced infarct volume in rats (Shyu et al., 2005) and PrP^C^-overexpressing mice showed smaller infarct volumes in response to ischemia than wild-type controls (Weise et al., 2008). However, another showed no difference in infarct sizes between wild-type and PrP^C^-overexpressing mice exposed to ischemia (Spudich et al., 2005). Of note, these studies used different PrP^C^-overexpressing mouse models and times of ischemia, which could account for the observed differences. Currently, the role of NMDA receptors in the enhanced ischemic cell death seen in the absence of PrP^C^ is unknown. However, because NMDA receptor hyperfunction contributes to cell death in ischemia (Lau and Tymianski, 2010; Lai et al., 2014), and since NMDA receptor desensitization is reduced in the absence of PrP^C^ (You et al., 2012), enhanced NMDA receptor-mediated Ca^2+^ influx may very well contribute to the increased ischemia-induced cell death that is observed in PrP^C^-null mice.

Increases in extracellular glutamate concentration, such as those seen in ischemic conditions, cause activation of both synaptic and extrasynaptic receptors (Hardingham and Bading, 2010; Parsons and Raymond, 2014). Extrasynaptic NMDA receptor activity can induce apoptotic signaling by promoting nuclear import of FOXO transcription factor proteins (Hardingham and Bading, 2010). FOXO3a has recently been identified as a negative regulator of the gene encoding PrP^C^, *prnp* (Liu et al., 2013), and in this study it was shown that insulin-like growth factor-1 (IGF-1)-induced PI3K/Akt activity promoted *prnp* expression by preventing nuclear import of FOXO proteins, thus inhibiting their negative effect on *prnp* expression. Therefore, it is possible that conditions where extrasynaptic NMDA receptor activity is upregulated may lead to a down-regulation of PrP^C^ protein expression, which may initiate a positive feedback loop of further dysregulation of NMDA receptor activity and thus toxicity. Hence, even if there is an initial protective upregulation of PrP^C^, this may not be able to be sustained due to persistent activation of extrasynaptic NMDA receptors in the presence of prolonged elevation of glutamate levels.

Another important observation concerns increased shedding of PrP^C^ in response to neurotoxic conditions (Wang et al., 2012). PrP^C^ shedding results from cleavage of PrP^C^ near the C-terminal GPI anchor, releasing full-length, soluble PrP^C^ into the extracellular milieu (Altmeppen et al., 2013; McDonald et al., 2014). Wang and colleagues showed that upon treatment of cultured rat cortical neurons with NMDA, soluble PrP^C^ was released into the culture medium, while total PrP^C^ protein levels remained unchanged (Wang et al., 2012). As noted earlier, our data demonstrated that treatment with PI-PLC, which cleaves PrP^C^ from its GPI anchor thus releasing it from the cell surface, resulted in persistent NMDA receptor-mediated current in cultured rat hippocampal neurons that was similar to those seen in PrP^C^-null hippocampal neurons (You et al., 2012). If loss of cell surface anchored-PrP^C^ results in aberrant NMDA receptor activity, one could envision a scenario where a positive feedback loop contributes to further shedding of PrP^C^ and enhanced NMDA receptor currents, which would be of detriment to cells. However, it has been suggested that PrP^C^ shedding could serve a protective role, especially in preventing Aβ-mediated toxicity (discussed in Altmeppen et al., 2013; Beland et al., 2014). A recent study by Beland and colleagues showed that shed PrP^C^ co-immunoprecipitated with Aβ (Beland et al., 2014), suggesting that extracellular Aβ can be sequestered by shed PrP^C^ to prevent Aβ-induced cellular damage. As discussed below, we found that Aβ_1-42_ induced a PrP^C^-dependent increase in steady-state NMDA receptor current (You et al., 2012). Shedding of PrP^C^ may thus aid in preventing Aβ _1-42_-induced dysregulation of NMDA receptor function. Hence, PrP^C^ shedding may ultimately be protective in situations where PrP^C^ function is compromised, for example by binding of Aβ peptides. On the other hand, shedding of PrP^C^ may promote aberrant NMDA receptor activity by altering desensitization kinetics that could contribute to cell death in conditions such as ischemia where glutamate concentrations are elevated.

There is also evidence that ischemia induces cleavage of PrP^C^ into its N- and C-terminal fragments, and that these cleavage products can be neuroprotective. Mitteregger and coworkers also observed that ischemia caused increased cleavage of PrP^C^, as detected by elevated levels of PrP^C^ C1 fragment on immunoblots of mouse brain homogenates (Mitteregger et al., 2007). In another study, oxygen glucose deprivation increased the secretion of the PrP^C^ N1 fragment from cultured retinal ganglion cells (Guillot-Sestier et al., 2009). Moreover, application of recombinant PrP^C^ N1 (PrP^C^ residues 23–110) was able to prevent ischemia-induced death of retinal ganglion cells (Guillot-Sestier et al., 2009). The details of how PrP^C^ N1 protects against ischemic cell death were not fully explored, but the mechanism did involve a reduction of ischemia-induced caspase-3 activation and p53 expression (Guillot-Sestier et al., 2009). An interesting possibility is that the PrP^C^ N1 fragment, which contains the octarepeat region that binds copper, binds to NMDA receptors, thus providing copper-dependent regulation of receptor desensitization. Of note, inhibition of NMDA receptor activity has been shown to attenuate death of retinal ganglion cells following ischemia-reperfusion injury (Li et al., 2014).

Further support of a role for PrP^C^ in protecting against ischemic cell death comes from the work of Mitteregger and coworkers where PrP^C^-null mice and mice expressing a prion protein lacking amino acid residues 32–93 had infarcts of similar volumes (Mitteregger et al., 2007), suggesting that this region of PrP^C^ provides protection against ischemic injury. One reason for increased ischemic injury in mice expressing this form of prion protein could be that cleavage of this mutant would not produce the normal PrP^C^ N1 fragment. However, this deletion mutant lacks the copper-binding octarepeat region of PrP^C^. As mentioned previously, we found a copper-dependent regulation of NMDA receptors that is mediated in large part through PrP^C^, as demonstrated by no further augmentation of NMDA receptor currents by copper chelation in the absence of PrP^C^ (You et al., 2012). Moreover, exogenous copper can protect neurons from NMDA receptor-mediated death (Schlief et al., 2006), whereas copper chelation induced cell death that could be prevented by NMDA receptor inhibition (You et al., 2012). Recently, data from *in vitro* experiments have revealed that copper enhances one type of α-cleavage, termed α2-cleavage, of mouse PrP^C^ (McDonald et al., 2014). Copper induced α2-cleavage such that the resultant N-terminal PrP^C^ fragments terminate at alanine residues 116 or 117, in contrast to α1-cleavage observed in the absence of copper, which produces PrP^C^ N1 ending at lysine residue 109 (McDonald et al., 2014). These observations suggest that copper levels may regulate PrP^C^ processing by influencing the amount and identity of the N1 fragment produced, although the biological outcomes of this copper-dependent differential PrP^C^ cleavage are not yet known. Of further interest is the finding that the prion proteins carrying pathogenic mutations have decreased α2-cleavage upon addition of copper (McDonald et al., 2014), suggesting aberrant copper-dependent PrP^C^ processing could be detrimental to the health of cells. These findings reveal the possibility of copper-dependent processing of PrP^C^ as another mechanism contributing to regulation of NMDA receptor activity by copper. Thus, copper may alter the activity of NMDA receptors in multiple ways: PrP^C^-independent control of receptor activity, as evidenced by restoration of NMDA receptor desensitization in PrP^C^-null neurons by addition of excess copper ions (You et al., 2012); by mediating interaction of PrP^C^ and NMDA receptors, as shown by a decrease in the amount of GluN1 subunit that co-precipitated with PrP^C^ upon copper chelation (You et al., 2012); and by influencing PrP^C^ cleavage to generate PrP^C^ N1 fragments, which could bind to NMDA receptors to modulate their function. Although evidence exists for the former two mechanisms, the latter remains to be investigated.

As mentioned previously, the activity of myelinic NMDA receptors is increased by ischemic conditions, resulting in damage to myelin (Micu et al., 2006). Thus, a loss of PrP^C^-mediated regulation of NMDA current may not only be neurotoxic, but may also contribute to demyelination that has been observed in PrP^C^-null mice and in mice expressing mutant prion proteins (Radovanovic et al., 2005; Baumann et al., 2007; Bremer et al., 2010).

## Aβ can disrupt PrP^C^-mediated regulation of NMDA receptor activity

PrP^C^ has been shown to be a receptor for oligomeric Aβ peptides (Lauren et al., 2009; Chen et al., 2010). Since these discoveries, many studies have investigated the role of PrP^C^ in the pathological effects of Aβ (reviewed in Lauren, 2014). Overall, these findings suggest a role for PrP^C^ in protecting from the pathological effects of Aβ peptides that occur in AD, although the requirement of PrP^C^ for Aβ-induced toxicity has been controversial, with some investigators finding that PrP^C^ was not required for the pathological effects of Aβ (Balducci et al., 2010; Calella et al., 2010; Kessels et al., 2010; Cisse et al., 2011). Recent findings by Nicoll and coworkers may help to reconcile these discrepancies. Their work showed that Aβ protofibrils, and not monomers or fibrils, bind with highest affinity to full-length PrP^C^ and the PrP^C^ N1 fragment (Nicoll et al., 2013). Furthermore, protofibril-rich Aβ preparations blocked LTP in a PrP^C^-dependent manner, whereas LTP inhibition by fibrillar Aβ was independent of PrP^C^. These observations underscore the need for careful characterization of Aβ preparations in order to determine the nature of the Aβ species used for experiments, which allow better interpretation of data resulting from these experiments.

**Figure 2:**
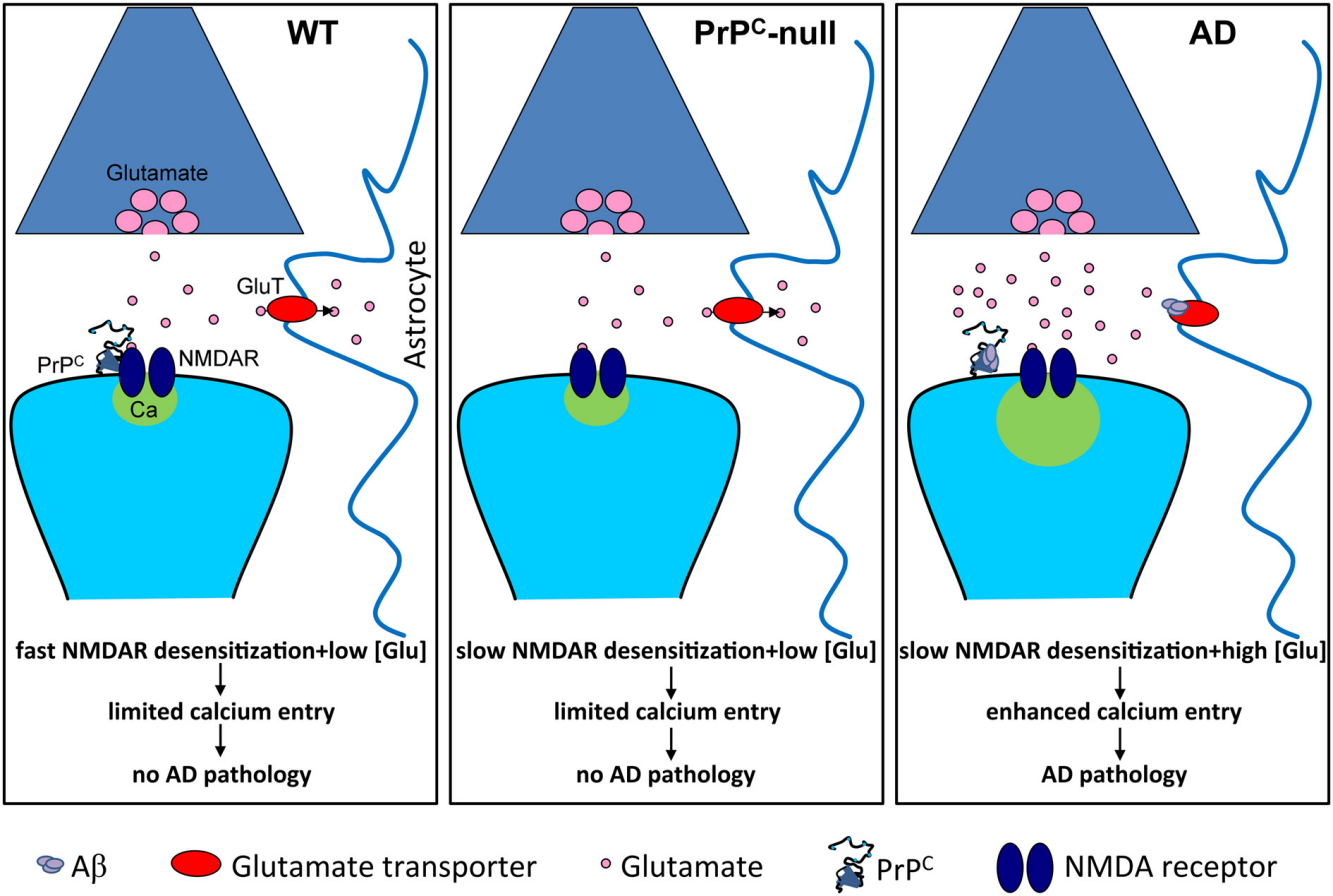
**Slowed NMDA receptor desensitization is pathological only in conditions of prolonged excess of glutamate**. Under normal conditions (WT), glutamate homeostasis is unperturbed and prevents glutamate accumulation, and NMDA receptors (NMDAR) undergo fast desensitization, thus limiting calcium entry. In the absence of PrP^C^ (PrP^C^-null), NMDA receptor desensitization is slowed but glutamate homeostasis remains unperturbed, thus limited calcium entry occurs. However, in Alzheimer's disease (AD), Aβ oligomers bind to PrP^C^ and cause slowed NMDA receptor desensitization. This, in combination with Aβ-induced elevation of glutamate levels, for example by inhibiting glutamate re-uptake by astrocytic glutamate transporters, leads to enhanced calcium entry and pathology.

Notably, many studies have demonstrated that Aβ can alter NMDA receptor function (reviewed in Rush and Buisson, 2014). Recently, we demonstrated that PrP^C^ is required for Aβ-induced alterations in NMDA receptor kinetics (You et al., 2012). Application of oligomerized Aβ_1-42_, which is known to be a high-affinity copper chelator, to hippocampal neurons gave rise to a persistent (i.e., non-desensitizing) NMDA current, similar to what was observed in the presence of the copper chelator BCS or in the absence of PrP^C^. Of note, the effects of Aβ_1-42_ and copper chelation on NMDA current were not additive, suggesting that they acted via the same mechanism. Finally, in neurons isolated from 5XFAD mice (a mouse model of AD), NMDA current displayed a qualitatively similar persistent component. Altogether, these data then suggest that Aβ-mediated perturbation of NMDA receptor activity depends on both PrP^C^ and copper ions. Furthermore, the Aβ_1-42_-induced upregulation of NMDA receptor activity may in part be responsible for the Aβ_1-42-mediated_ neurodegeneration observed in AD. If this were the case, then why is neurodegeneration observed in 5XFAD mice, but relatively absent in PrP^C^-null mice even though they both display similar non-desensitizing NMDA receptor currents? Slowed receptor desensitization can only manifest itself pathologically under conditions where there is a prolonged excess of glutamate. It has been shown that Aβ_1-42_ prevents glutamate re-uptake into neurons and glial cells (Danysz and Parsons, 2012), and induces release of glutamate from astrocytes (Talantova et al., 2013), which then would be expected to lead to just such an excess of glutamate. Then, together with the slowed desensitization kinetics of the receptor, this excess glutamate may damage neuronal structures such as spines. In PrP^C^-null mice, this accumulation of glutamate would not be expected to occur, thus rendering the slowed desensitization kinetics physiologically inert (Figure 2). Nonetheless, as we discussed earlier, there are other pathophysiological events such as chemically-induced seizures or ischemia that are known to lead to an accumulation of glutamate that then causes increased brain damage in PrP^C^-null mice (Walz et al., 1999; McLennan et al., 2004; Sakurai-Yamashita et al., 2005; Spudich et al., 2005; Weise et al., 2006; Mitteregger et al., 2007; Steele et al., 2009).

Work by Um and colleagues provides further evidence for the requirement of PrP^C^ on the effects of Aβ on NMDA receptor function (Um et al., 2012). In their study, they found that phosphorylation of the GluN2B subunit of the NMDA receptor at tyrosine residue 1472 was transiently increased by Aβ oligomers in Fyn-overexpressing neuroblastoma cells and in cortical cultures; longer treatment with Aβ oligomers (1–3 h) resulted in decreased phospho-GluN2B levels, which correlated with an increase in the level of STEP protein phosphatase. Moreover, the Aβ oligomer-induced increase in GluN2B phosphorylation was absent in PrP^C^- and Fyn-null cultures. Indeed, they found that Aβ oligomers activated Fyn kinase in a PrP^C^-dependent manner. Furthermore, brain extract from AD patients induced Fyn activation in cultured mouse cortical neurons in a PrP^C^-dependent manner, whereas extract from healthy controls did not activate Fyn. The initial GluN2B phosphorylation was paralleled by an increase in surface levels of GluN2B, and with longer Aβ oligomer treatment a reduction in GluN2B surface expression was observed; again, both PrP^C^ and Fyn were required for these Aβ oligomer-mediated effects on GluN2B surface expression. In cultures from wild-type mice, pre-treatment for 15 min with Aβ oligomers significantly enhanced the NMDA-induced increase in intracellular Ca^2+^ levels, whereas a 60 min Aβ oligomer pre-treatment significantly reduced the NMDA-induced Ca^2+^ signal; in PrP^C^-null cultures, Aβ oligomers had no effect on the Ca^2+^ signal induced by NMDA. Thus, Aβ oligomers, acting via PrP^C^, induce a transient increase then a decrease in NMDA receptor-mediated Ca^2+^ influx into neurons. LDH release from cortical cultures was increased upon 90 min Aβ oligomer treatment and no further toxicity was observed with a 72 h treatment. This toxicity was NMDA receptor-dependent, as it was reduced by NMDA receptor inhibition with both APV and ifenprodil, the latter being a specific GluN2B antagonist. Thus, brief exposure to Aβ oligomers causes toxicity, likely by Ca^2+^ influx mediated by the transient increase in surface localization of GluN2B-containing NMDA receptors. In this study, it was also shown that Aβ oligomers induced spine loss in a PrP^C^- and Fyn-dependent manner, and that the seizures and death due to status epilepticus in a mouse model of AD were prevented by genetic deletion of PrP^C^. Taken together, these data suggest that Aβ oligomers signal through PrP^C^ to induce aberrant Fyn activation, leading to first an increase then later a decrease in activity of GluN2B-containing receptors, which is ultimately deleterious to neurons. Altogether, the recent data indicate that Aβ oligomers, by interacting with PrP^C^, can alter both NMDA receptor kinetics (You et al., 2012) and the signaling pathways that modulate NMDA receptor function (Um et al., 2012).

A study by Resenberger and colleagues showed that Aβ- and PrP^Sc^-induced cell death of PrP^C^-expressing SH-SY5Y neuroblastoma cells were not additive, suggesting that these species act via the same pathway (Resenberger et al., 2011). Furthermore, pre-treatment with the NMDA receptor blocker memantine prevented apoptosis of PrP^C^-expressing SH-SY5Y neuroblastoma cells induced by PrP^Sc^, Aβ, and a synthetic peptide designed to be rich in β-sheet structure. This suggests that several types of β-sheet-containing proteins/peptides share a common mechanism of toxicity, aberrant NMDA receptor activity. Thus, PrP^C^ could contribute to toxicity in disorders other than TSEs and AD, possibly by mediating aberrant NMDA receptor activity in the presence of β-sheet-rich peptides.

Of further interest is the finding that the toxicity induced by β-sheet-rich conformers was dependent upon the N-terminal region of PrP^C^ and was prevented by a soluble N-terminal domain of PrP^C^ (Resenberger et al., 2011). Similar protective effects of the PrP^C^ N1 fragment against Aβ-induced toxicity have been observed in other studies (Beland et al., 2012, 2014; Guillot-Sestier et al., 2012; Nieznanski et al., 2012; Fluharty et al., 2013). This raises the possibility that increased PrP^C^ cleavage to produce the N1 fragment is neuroprotective. Interestingly, cleavage of PrP^C^ was increased in a mouse model of AD (Ostapchenko et al., 2013) and in post-mortem human AD brain (Beland et al., 2014). Furthermore, the recent findings from Beland and coworkers provide strong evidence for a protective role of the soluble N1 fragment that is produced by α-cleavage of PrP^C^. In this study, the binding of PrP^C^ N1 and Aβ induced a conformational change that produced amorphous aggregates, the amount of PrP^C^ N1 in guanidine hydrochloride extracts from insoluble amyloid deposits from AD brain was increased compared to non-demented controls, and there was a significant correlation between the amount of α-cleavage, which generates the N1 fragment, and duration of AD (Beland et al., 2014). Altogether, these observations indicate that cleavage of PrP^C^ may reduce Aβ-mediated toxicity via N1-mediated neutralization of Aβ peptides, and N-terminal fragments may be useful as a therapy to prevent pathological effects of Aβ or other β-sheet-rich peptides, including alterations in NMDA receptor activity.

## NMDA receptors, PrP^C^, and neuroinflammation

Recent evidence has emerged that glutamatergic mechanisms operate not only in the nervous system, but also in a wide variety of non-neuronal cells (reviews: Gill and Pulido, 2001; Skerry and Genever, 2001; Hinoi et al., 2004), including in the immune system (reviews: Boldyrev et al., 2005, 2012). GluRs, both ionotropic and metabotropic, are highly expressed in various immune cells, such as T cells, B cells, neutrophils, macrophages and dendritic cells, which are thought to subserve communication between the immune and nervous systems (review: Ganor and Levite, 2012). Interestingly, different GluRs, or different levels of certain GluRs, are expressed in resting and activated T cells (for review see Ganor and Levite, 2012). In particular, NMDA receptors expressed on T cells are involved in a wide variety of T cell functions, such as regulation of cytokine secretion (Kahlfuβ et al., 2014), proliferation (Kahlfuβ et al., 2014), apoptosis (Affaticati et al., 2011), and induction of reactive oxygen species (Tuneva et al., 2003).

As described above, PrP^C^ interacts with NMDA receptors on neurons and modulates NMDA receptor-dependent neuronal excitability and excitotoxicity (Khosravani et al., 2008). Although the precise functions of PrP^C^ in immune cells remain unclear, PrP^C^ expression is detected in the lymphoid system (Bendheim et al., 1992; Ford et al., 2002). Human T lymphocytes constitutively express PrP^C^ and its surface expression is influenced by the activation state of the cells (Cashman et al., 1990; Mabbott et al., 1997). In addition, PrP^C^ is upregulated on mouse T cells and dendritic cells after activation, and the lack of PrP^C^ increases T cell proliferation and causes T cell over-activation in mouse (Tsutsui et al., 2008). Therefore, PrP^C^ may play a role as a regulator of NMDA receptors in the immune system as we observed in the nervous system (Khosravani et al., 2008; You et al., 2012), with a common thread being “hyperfunction” when PrP^C^ is absent and cannot restrain normal physiological mechanisms.

The CNS is conventionally recognized as being “immunologically privileged” (Bailey et al., 2006) and is anatomically separated from the peripheral immune system by the presence of the blood-brain barrier (BBB) (Xiao and Link, 1998; Weller et al., 2009). Despite the presence of the BBB, which limits the entry of cells and pathogens to the brain, lymphocytes can traffic into the CNS to surveil the local environment (Xiao and Link, 1998; Kivisakk et al., 2003). It is increasingly recognized that the CNS is capable of shaping the immune response (for review see Xiao and Link, 1998); it is now clear that neuroinflammation is a well-established hallmark of a number of neurodegenerative diseases, including AD, and is frequently detrimental to neurological function (Akiyama et al., 2000; Rubio-Perez and Morillas-Ruiz, 2012; Solito and Sastre, 2012; Cappellano et al., 2013; Enciu and Popescu, 2013; Lynch, 2014). In the AD brain, the inflammatory response is thought to be a secondary response caused by an initial brain insult, which is provided by damaged neurons, neurites, insoluble Aβ aggregates, and neurofibrillary tangles as stimuli (Akiyama et al., 2000). However, recent evidence suggests that inflammatory mediators may stimulate amyloid precursor protein processing, and therefore, play a role as a driving force to establish a deleterious cycle to AD progression (Heneka et al., 2010). As described above, PrP^C^ is a receptor for oligomeric Aβ peptides (Lauren et al., 2009; Chen et al., 2010; Lauren, 2014), and Aβ oligomers, acting via PrP^C^, modulate NMDA receptor-mediated Ca^2+^ influx into neurons. Perhaps, even soluble forms of small oligomers, may directly stimulate PrP^C^ and modulate NMDA receptor-mediated Ca^2+^ influx into immune cells to trigger signaling pathways to secrete cytokines, reactive oxygen species, and/or regulate T cell polarization at the early stage of AD. Gaining more insight into the role of PrP^C^-NMDA receptor interactions in neuroinflammation may reveal novel approaches to visualize early AD pathogenesis and diagnosis.

## Concluding remarks

It is becoming clear that loss of PrP^C^ regulation of NMDA receptors can result in toxicity in a variety of pathological conditions, and that Aβ can cause aberrant activation of NMDA receptors in a PrP^C^-dependent manner. The possibility of restoring the NMDA receptor-PrP^C^ interaction as a way to protect against excitotoxicity will need to be tested experimentally, and in order to accomplish this, elucidation of the molecular details of the interaction between PrP^C^ and NMDA receptors is needed. In light of the recent findings regarding cleavage of PrP^C^ and how this is altered in disease, there is a need for investigation of how physiological processing of PrP^C^ impacts NMDA receptor activity in both health and disease. Overall, a better understanding of the molecular mechanisms that determine how PrP^C^ regulates NMDA receptor function will open up new therapeutic avenues in situations where the regulation of NMDA receptors by PrP^C^ is perturbed, such as in AD and prion diseases.

### Conflict of interest statement

The authors declare that the research was conducted in the absence of any commercial or financial relationships that could be construed as a potential conflict of interest.
